# Augmented reality with image registration, vision correction and sunlight readability via liquid crystal devices

**DOI:** 10.1038/s41598-017-00492-2

**Published:** 2017-03-27

**Authors:** Yu-Jen Wang, Po-Ju Chen, Xiao Liang, Yi-Hsin Lin

**Affiliations:** 1 Department of Photonics, National Chiao Tung University, Hsinchu, Taiwan; 2 Department of Chemistry, Tsinghua University, Beijing, China

## Abstract

Augmented reality (AR), which use computer-aided projected information to augment our sense, has important impact on human life, especially for the elder people. However, there are three major challenges regarding the optical system in the AR system, which are registration, vision correction, and readability under strong ambient light. Here, we solve three challenges simultaneously for the first time using two liquid crystal (LC) lenses and polarizer-free attenuator integrated in optical-see-through AR system. One of the LC lens is used to electrically adjust the position of the projected virtual image which is so-called registration. The other LC lens with larger aperture and polarization independent characteristic is in charge of vision correction, such as myopia and presbyopia. The linearity of lens powers of two LC lenses is also discussed. The readability of virtual images under strong ambient light is solved by electrically switchable transmittance of the LC attenuator originating from light scattering and light absorption. The concept demonstrated in this paper could be further extended to other electro-optical devices as long as the devices exhibit the capability of phase modulations and amplitude modulations.

## Introduction

When human lifespan increases, people would naturally increase a great demand for high quality of life during old age as well as a desire to live healthier for longer. According to United Nations (UN), the ageing work force is already a global phenomenon and UN suggests that the older work force should be permitted to work as long as they want and they are capable of being productive^1, 2^. However, people face the descending problems physiologically and mentally as people get old. To extend mobility and fight diseases of old age, “bionic people” defined as people with augmented electronics is a trend and many electronic devices are developing to augment hearing, memory, vision or other human faculties^2^. Augmented reality (AR) is a technology that visually augments the real-world environment by projecting computer-generated information to eyes^3–7^. Such a technology could be exploited in bionic people with augmented memory or vision, and even applications of neuroscience therapy^2, 8^. M. Mengoni, *et al*. proposed a usage of AR in monitor-based device to provide user-friendly interface for ageing workers^9^. The wearable type of AR could even assist workers who need their both hands free while working. Many types of wearable AR are developing and we focus on optical see-through type in this paper^10^. The optical see-through type of wearable AR consists of a liquid crystal display (LCD) panel, projection lens modules, and light guides in general. Three major challenges of optical see-through type of AR stay years to hinder the practical applications^10^. First challenge is registration problem which means the projected virtual image is not coincide with the physical object due to fixed optical properties of optical elements. The discrepancy between the virtual image and the physical object results in visual fatigue because people have to continuously adjust the crystalline lenses in eyes for accommodation. To solve this, lenses with electrically tunable lens powers (liquid lens or liquid crystal lenses) could help to adjust the location of projected virtual images^11, 12^. The second challenge is vision correction. People have various eye conditions, such as myopia, hyperopia, presbyopia, astigmatism and so on. According to statistics, at least 23.4% of Americans and 42.1% of Taiwanese above age 40 suffer from myopia and the number is still increasing^13, 14^. Aged people have presbyopic eye conditions due to aged crystalline lenses of eyes. Fixed optical properties of AR system cannot satisfy all the eye conditions which would lower comforts of users. It is necessary to add a lens with a tunable lens power for all kinds of eye conditions without conventional prescription lens. The third challenge is readability of projected virtual images under strong ambient light. In optical see-through AR systems, the projected virtual image is not opaque which leads to the projected virtual images (or overlaid graphics) cannot completely obscure the physical objects behind them. As a result, the superimposed text (i.e. virtual image) may be hard to read against some backgrounds. The contrast decrease of the virtual image (or image washout) under strong ambient light is predictable. R. Zhu *et al.* proposed functional reflective polarizer in AR system to solve readability problem and also proposed a combination of reflective polarizer and tunable liquid crystal (LC) film to adjust the intensity of ambient light^15, 16^. In addition, H. Chen *et al.* proposed a method by a usage of a cycloidal diffractive waveplate to improve user viewing experience in AR^17^. To date, no proposed AR system could solve three major challenges simultaneously. Those challenges therefore motivate us to develop an AR system which could solve three challenges at once: registration, vision correction, strong light readability via electrically tunable LC devices. Previously, we demonstrated an AR system with a single LC lens for adjusting virtual images in order to solve registration^12^. In this paper, we solve three challenges of optical see-through AR system simultaneously for the first time by demonstrating an AR system with three LC devices: two LC lenses with electrically tunable lens powers and one polarizer-free LC attenuator with electrically tunable attenuation. One LC lens is responsible for harnessing location of virtual image to solve registration. The other LC lens is in charge of vision correction, including myopia, hyperopia, and presbyopia. The lens powers of two LC lenses exhibit some linearity. The polarizer-free LC attenuator based on light scattering and light absorption is exploited for contrast enhancement of images under strong ambient light. The operating principles of the whole system and individual LC device are introduced and the experiments are performed for proof-of-concept. The concept proposed is not only limited to specific LC devices demonstrated in this paper, but also could be extended to many kinds of optical devices as long as the devices could be operated in phase-only modulations and amplitude modulations. The AR system we proposed paves a way to future bionic people with augmented vision and memory.

## Operating principles

**Figure 1:**
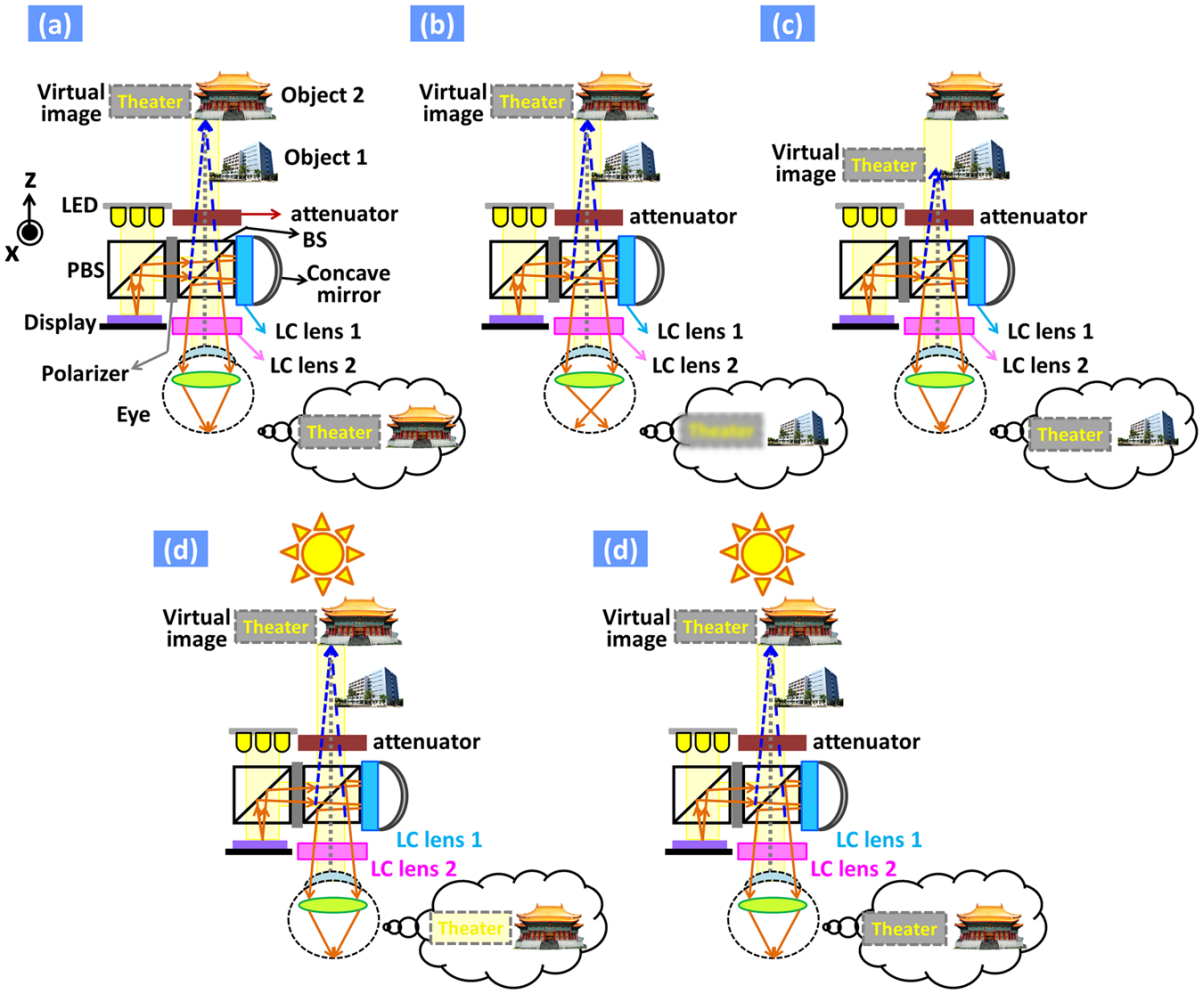
Operating principles of the AR system. (**a**) Initially, “LC lens 1”, “LC lens 2”, and attenuator are off. People see the “object 2” and the virtual image “Theater” projected from the display. (**b**) When “LC lens 2” turns on and provides a positive lens power to the system, people see the “object 1”, but the virtual image is blurred. (**c**) From (**b**), the “LC lens 1” then turns on and provides a positive lens power, the virtual image is shifted to the location of “object 1”. People see both of the virtual image and “object 1”. (**d**) From (**a**), the virtual image is washed out under strong ambient light. (**e**) From (**d**), people can see the virtual image when the attenuator turns on. The “object 1” is photographed by Pi Cheng Wu and the copyright is released to the authors and Nature Publishing Group. The “object 2” is photographed by Fred Hsu and distributed under license of CC BY-SA 3.0 (https://creativecommons.org/licenses/by-sa/3.0/) on https://en.wikipedia.org/wiki/National_Theater_and_Concert_Hall,_Taipei#/media/File:Taiwan_2009_Taipei_National_Theater_at_Chian_Kai_Shek_Cultural_Center_FRD_7363_Pano_Extracted.jpg.

The AR system we proposed is depicted in Fig. 1(a–d). Light from LED travels to reflective LCoS panel and the image on the LCoS panel is then reflected to polarizing beam splitter (PBS), polarizer, a beam splitter (BS), “LC lens 1” and a concave mirror. Thereafter, the image is reflected by the concave mirror and goes to “LC lens 1”, BS, and “LC lens 2”. Finally, the image arrives in human eyes in which light keeps traveling to the cornea, crystalline lens, and retina. As a result, eyes see the virtual image, projected from LCoS panel; meanwhile, eyes see the objects in the real world through the viewing window (i.e. BS in Fig. 1(a)). This is the so-called AR, eyes see the real world augmented by the projected virtual image. For myopic and presbyopic eyes, for example, people see “object 2” clearly in Fig. 1(a), but not objects at other locations. Initially, “LC lens 1”, “LC lens 2”, and attenuator are off. People see the “object 2” and the virtual image “Theater” projected from the LCoS panel (Fig. 1(a)) when properly setting up the AR system. When “LC lens 2” turns on to correct the vision by providing a positive lens power, people see the “object 1”. However, the virtual image is blurred (Fig. (b)). In order to see both of the virtual image and “object 1”, the “LC lens 1” then turns on to add a positive lens power. The virtual image is then shifted to the location of “object 1” (Fig. (c)). This is so-called the registration of images. Here, we take advantage of the polarized light that reflected from LCoS, so that the “LC lens 1” is polarization dependent and we could double the lens power due to reflection of light from the concave mirror. From Fig. 1(a), the virtual image is washed out under strong ambient light as shown in Fig. 1(d). People can see the virtual image with better visibility when the attenuator turns on (Fig. 1(e)). Briefly, “LC lens 1” is responsible for registration which means adjustment of virtual image, “LC lens 2” is in charge of vision correction by adding positive or negative lens powers, and the LC attenuator is exploited for contrast enhancement of virtual images under strong ambient light.

To prove the proposed AR system, we define *d*_*vision*_ as a distance between the real object and “LC lens 2”. *d*_*vision*_ is related to effective lens power of eyes *S*(*P*_*cryst*_) and lens power (*P*_*LC2*_) of “LC lens 2” is expressed as^18^:

1 $${{d}}_{vision}({{P}}_{LC2}({V}),\,{S}({{P}}_{cryst}))=\frac{1}{{{P}}_{LC2}(V)+{S}({{P}}_{cryst})}$$

where *P*_*cryst*_ is the lens power of the crystalline lens and *V* is applied voltage. *S*(*P*_*cryst*_) is limited by the near point and the far point which are the closest and farthest object points for the clear vision, respectively. Assume that the distance between the far point and eyes is *d*_*far*_ and the distance between near point and eyes is *d*_*near*_. As a result, the range of *S*(*P*_*cryst*_) should be: $1/{{d}}_{far}\le {S}({{P}}_{cryst})\le 1/{{d}}_{near}$. The distance between the far and near points is called the range of accommodation. Assume *P*_*LC2*_ is also limited in a certain range: ${{P}}_{LC2,{\rm{\min }}}\le {P}_{LC2}\le {P}_{LC2,{\rm{\max }}}$. As a result, ${d}_{near}/({d}_{near}\times {P}_{LC2,{\rm{\max }}}+1)\le {d}_{vision}\le {d}_{far}/({d}_{far}\times {p}_{LC2,{\rm{\min }}}+1)$ according to Eq. (1) and the constraints of *S*(*P*_*cryst*_) and *P*_*LC2*_. This indicates that *P*_*LC2*_ could correct vision by harnessing the range of *d*_*vision*_. Thereafter we further discuss how “LC lens 2” correct in vision. When effective lens power of eye is 1/*d*_*far*_ (i.e. *S*(*P*_*cryst*_) = 1/*d*_*far*_), the negative lens power of “LC lens 2” (i.e. *P*_*LC2*_(*V*) < 0) could help people to see further and d_vision_ is equal to ${d}_{far}/({P}_{LC2}(V)\times {d}_{far}+1)\ge {d}_{far}$. Similarly, when *S*(*P*_*cryst*_) = 1*/* *d*_*near*_, the positive lens power of “LC lens 2” (i.e. *P*_*LC2*_(*V*) > 0) could help people to see nearer and then *d*_*vision*_ is equal to ${d}_{near}/({P}_{LC2}(V)\times {d}_{near}+1)\ge {d}_{near}$. When *P*_*LC2*_(*V*) = 0, d_vision_ = 1/*S*(*P*_*cryst*_). How *P*_*LC2*_(*V*) changes the far point and near point of vision is listed in Eq. (2).

2 $${d}_{vision}=\{\begin{array}{l}{d}_{far}/({P}_{LC2}(V)\times {d}_{far}+1)\ge {d}_{far},\\ 1/S({P}_{cryst}),\\ {d}_{near}/({P}_{LC2}(V)\times {d}_{near}+1)\le {d}_{near},\end{array}\,\begin{array}{c}{P}_{LC2}(V)\le 0,S({P}_{cryst})=1/{d}_{far}\\ {P}_{LC2}(V)=0\\ {P}_{LC2}(V)\ge 0,S({P}_{cryst})=1/{d}_{near}\end{array}.$$

In Eq. (2), people with different eye conditions(i.e. different *S*(*P*_*cryst*_)) indicate different *d*_*far*_ and *d*_*near*_. However, “LC lens 2” could help people to keep the same *d*_*vision*_ as long as we change the corresponding lens power of “LC lens 2” by applying voltages. The “LC lens 2” functions as an augmented accommodation for eyes. In addition, the location of projected virtual image is also adjustable. Assume *d*_*image*_ is the distance between projected virtual image and “LC lens 2”. According to Fig. 1(a) and image formation, voltage-dependent *d*_*image*_ is^12^:

3 $${d}_{image}(V)=\frac{-1}{2\times {P}_{LC1}(V)+{P}_{mirror}-\frac{1}{{d}_{system}}}-{d}_{1}+{d}_{2}$$

where *P*_*LC*1_ is the lens power of “LC lens 1”, *P*_*mirror*_ is the lens power of the concave mirror, *d*_*system*_ is the effective optical path between LCoS panel and “LC lens 1’, *d*_1_ is distance between BS and “LC lens 1”, and *d*_2_ is a distance between BS and “LC lens 2”. This indicated the location of virtual image is harnessed by electrically controlling the lens power of “LC lens 1”. Thus, people could view both of a virtual image and the real object in focus at the same time no matter where the object is, which means the registration is solved. From Eqs (1) and (3), both of the vision correction and registration are satisfied under a condition: ${d}_{vision}({V}_{LC2})={d}_{image}({V}_{LC1})$. Hence, it leads to the relation between *P*_*LC1*_ and *P*_*LC2*_:

4 $${P}_{LC2}(V)=\frac{2\times {P}_{LC1}(V)+{P}_{mirror}-\frac{1}{{d}_{system}}}{({d}_{2}-{d}_{1})(2\times {P}_{LC1}(V)+{P}_{mirror}-\frac{1}{{d}_{system}})-1}-S({P}_{cryst}).$$

In Eq. (4), *d*_1_~*d*_*2*_ > 0. Eq. (4) could be simplified as Eq. (5).

5 $${P}_{LC2}(V)+S({P}_{cryst})=-2\times {P}_{LC1}(V)-({P}_{mirror}-\frac{1}{{d}_{system}})$$

The location of the projected virtual image is adjusted by *P*_*LC*1_(*V*). Assume the range of *P*_*LC*1_(*V*) is ${P}_{\min }\le {P}_{LC1}(V)\le {P}_{\max }$. The range of *P*_*LC*1_(*V*) constrains the range of *P*_*LC*2_(*V*):

6 $$(1/{d}_{system}-{P}_{mirror})-2\times {P}_{\max }\le {P}_{LC2}(V)+S({P}_{crystal})\le (1/{d}_{system}-{P}_{mirror})-2\times {P}_{\min }$$

Thus, the vision correction and registration in an AR system could be achieved simultaneous by altering lens powers of two LC lenses when the inequality holds. The readability under strong ambient light in AR system is poor resulting from the projected virtual images are not opaque. As a result, lower ambient light intensity to improve the contrast is a solution. Electrically tunable LC attenuators are good candidates for the purpose. The mechanism of light attenuation in this paper is based on scattering and absorption. We will introduce the detail structure in the next section.

**Figure 2:**
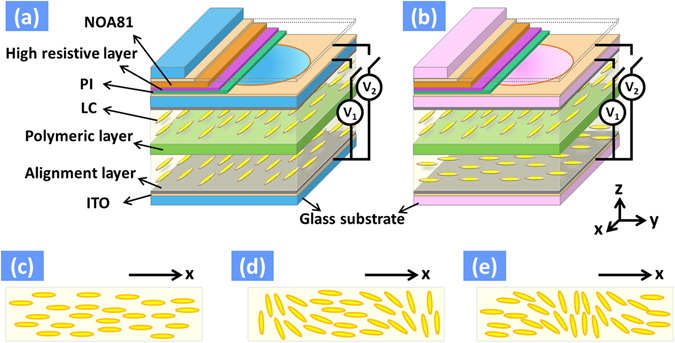
The structure of (**a**) “LC lens 1” in Fig. 1(a) and (b) “LC lens 2” in Fig. 1(a). A top LC layer of (**a**) and (**b**) at (**c**) *V*_1_ = *V*_2_ = 0, (**d**) *V*_1_ > *V*_2_, and (**e**) *V*_1_ < *V*_2_. The bottom LC layer of (**a**) is identical to top LC layer and the bottom LC layer of (**b**) is orthogonal in orientations to the top LC layer.

Generally speaking, the LC lens is operated under non-uniform electric fields to create spatial distribution of refractive index, which introduce the spatial phase distribution equivalent to that of conventional lens. To achieve augmented reality system with vision correction, the LC lenses are required to have the larger aperture size (>10 mm) and three ways in general: one is the Fresnel zone electrode structure^19–21^, the second one is a passive anisotropic element combined with a LC polarization switching device^22–24^, the third one is hole pattern electrode structure^25, 26^. However, the diffraction efficiency and image quality of Fresnel type LC lens due to ring electrodes are quite challenge. The one for a passive anisotropic element combined with a LC polarization switching device requires polarizer. Here, we use hole-patterned electrode structure of LC lenses. The structures of two LC lenses are shown in Fig. 2(a) and (b). The “LC lens 1” in Fig. 1(a) is polarization dependent because the light reflected from LCoS panel (or light for the virtual image) is linearly polarized. On the contrary, “LC lens 2” is polarization independent because “LC lens 2” is used for vision correction while people see the environment. Figure 2(a) and (b) illustrate two LC lenses at *V*_1_ = *V*_2_ = 0, where *V*_1_ and *V*_2_ are denoted as voltages applied to the hole-patterned electrode and flattened electrode, respectively. Figure 2(c–e) represent top LC layers of Fig. 2(a) and (b) at different voltages. The bottom LC layer of Fig. 2 (a) is identical to top LC layer and the bottom LC layer of Fig. 2(b) is orthogonal in orientations to the top LC layer. The lens powers of two LC lenses are zero at *V*_1_ = *V*_2_ = 0. When *V*_1_ > *V*_2_, the electric field in the periphery of aperture is stronger than the one in the center of aperture; therefore, the LC molecules near the periphery of the hole-patterned electrode are more perpendicular to the glass substrate than those in the center (Fig. 2(d)). When *V*_1_ < *V*_2_, hole-patterned electrode provides a weaker electric field than flat electrode does, which makes the LC molecules in the center of aperture more perpendicular to the glass substrate than those in the periphery of aperture (Fig. 2(e)). Assume an unpolarized incident light propagates along + z-direction and its random polarization (${\mathop{E}\limits^{\rightharpoonup }}_{i}$) can be decomposed as x- and y-linearly polarized light with randomly relative phase difference^26^:

7 $${\mathop{E}\limits^{\rightharpoonup }}_{i}={A}_{x}\cdot \hat{x}+{A}_{y}\cdot \hat{y},$$

where *A*_*x*_ and *A*_*x*_ are complex number; $\hat{x}$ and $\hat{y}$ are the unit vector along +x and +y direction. When light passes through two orthogonal LC layers in Fig. 2(b), two eigen-polarizations (i.e. x- and y-linearly polarized light) experience same phase shift. Thereafter, light propagate out of the LC lens (${\mathop{E}\limits^{\rightharpoonup }}_{out}$) can be described as^26^.

8 $${\mathop{E}\limits^{\rightharpoonup }}_{out}={e}^{j2\times k\times d\times ({n}_{o}+{n}_{c})}\cdot {e}^{+j2\times k\times d\times \frac{{n}_{b}-{n}_{c}}{{{r}_{0}}^{2}}\times {r}^{2}}\cdot {\mathop{E}\limits^{\rightharpoonup }}_{i},$$

where *k* is the wave number, d is the thickness of a LC layer, *r* is $\sqrt{{x}^{2}+{y}^{2}}$, and *r*_0_ is radius of the LC lens. *n*_*e*_ and *n*_*o*_ are extraordinary and ordinary refractive index of LC, respectively. *n*_*b*_ and *n*_*c*_ are central and peripheral refractive index of the LC lens, respectively. The first exponential term is absolute phase and the second exponential term in Eq. (8) is phase term for lensing effect. Under thin lens approximation, the second exponential term approximately equals to phase transformation of ${e}^{-jk\times \frac{{r}^{2}}{2\times f}}$, where *f* is focal length. As a result, the lens power of the “LC lens 2”, an inverse of focal length, in Fig. 2(b) is^26^:

9 $${P}_{LC}(V)=\frac{2\cdot \delta n(V)\cdot d}{{{r}_{0}}^{2}},$$

where *δn* is the difference of refractive indices as the LC in the central and peripheral region (i.e. *n*_*c*_ − *n*_*b*_). The lens power of “LC lens 2” is half of “LC lens 1” because of only one effective LC layer of “LC lens 2” to contribute the lens power.

**Figure 3:**
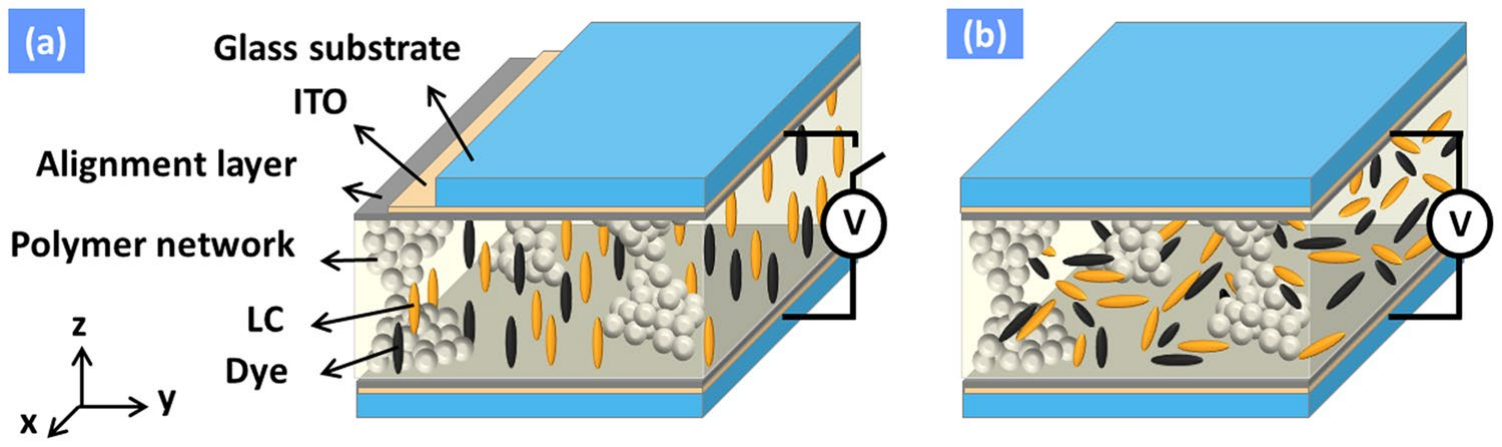
The operating principles of LC attenuator at (**a**) voltage-off state and (**b**) voltage-on state.

The LC attenuator is depicted in Fig. 3(a). To prove the concept, the structure of the LC attenuator is based on guest-host LC gels^27^. The LC attenuator consists of LC molecules, dichroic dye molecules, and polymer networks. When the linearly polarized light oscillates along long axes of dye molecules, the light is strongly absorbed. Otherwise, the light is weakly absorbed as the linearly polarized light oscillates along short axes of dye molecules. At voltage-off state in Fig. 3(a), LC molecules, dye molecules and polymer networks are along z-direction. When unpolarized light propagates along z-direction, the light experiences little absorption and little scattering. At voltage-on state or the voltage exceeds certain voltage, the LC molecules randomly tilt away from z-direction due to negative dielectric anisotropy. In addition, the dye molecules are reoriented by LC molecules. When the unpolarized light along z-direction enters to the LC attenuator, the light is absorbed and scattered. The light absorption and scattering reach maximum when the LC molecules and dye molecules are randomly distributed along x-y plane. By properly adjusting the domain size of polymer networks, the scattering could be reduced. The light absorption and scattering are polarization independent which indicates the LC attenuator is polarizer-free.

## Results and Discussion

**Figure 4:**
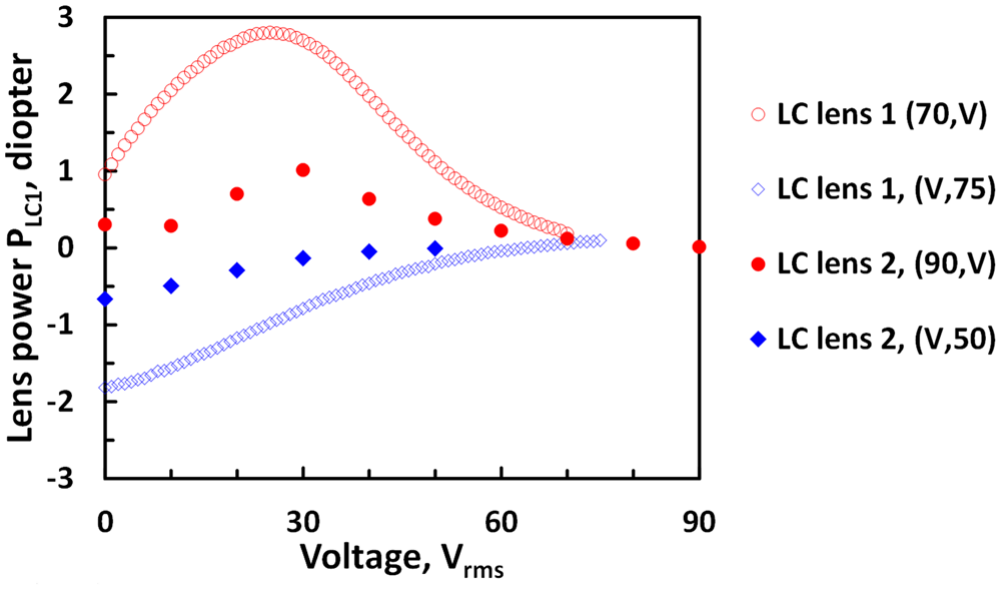
Voltage-dependent lens power of “LC lens 1” and “LC lens 2”. Red circles stand for lens power of “LC lens 1” at a fixed *V*_1_ of 70 *V*_rms_ while at different *V*_2_ (*f* = 7 kHz), and blue hollow diamonds stand for lens power of “LC lens 1” at different *V*_1_ while at a fixed *V*_2_ of 75 V_rms_ (*f* = 2 kHz). Red dots stand for lens power of “LC lens 2” at a fixed *V*_1_ of 90 V_rms_ while at different *V*_2_ (*f* = 3.75 kHz), and blue diamonds stand for lens power of “LC lens 2” at different *V*_1_ while at a fixed *V*_2_ of 50 V_rms_ (*f* = 0.4 kHz). The apertures of “LC lens 1” and “LC lens 2” are 4 mm and 10 mm, respectively.

The lens powers as a function of voltage (*V*_1_, *V*_2_) of two LC lenses are shown in Fig. 4. Both of the lens power of “LC lens 1” and “LC lens 2” are electrically tunable. The measured lens power of “LC lens 1” with aperture of 4 mm ranges from −1.82 D to +2.80 D while “LC lens 2” with aperture of 10 mm is from −0.67 D to +1.01 D. Since the LCoS panel is approximately 4 mm in diagonal, we only used the measurement result of the “LC lens 1” with the effective aperture size of 4 mm, even though the whole aperture of “LC lens 1” are 10 mm and the lens power is from −2.37 D to +1.56 D. Under the same aperture size, the tunable range of lens powers in “LC lens 1” is larger than “LC lens 2” which agrees with the previous discussion. In addition, the maximum lens power occurs when *Δn* in Eq. (5) approximately equals to birefringence of LC (i.e. *Δn* = 0.367). As a result, the tunable range of “LC lens 1” (aperture of 10 mm) theoretically should be −2.07D~+2.07D which is larger than experimental results (−2.37D to +1.56 D). As to the “LC lens 2”, the theoretical lens power should be in a range of −1.03D to +1.03D which is also larger than experimental results (−0.67D to +1.01D). The smaller tunable range of lens powers in experiments might result from two reasons. One is because the maximum lens power in Eq. (9) is not achieved as the LC molecules in the aperture center actually could tilt up. The other is the applied electric fields across to two LC layers are not identical due to asymmetry of patterned electrodes^28^ (i.e. the bottom planar electrode and the hole-patterned electrode) and then result in polarization dependency which could reduce effective lens power in Eq. (8). Moreover, two LC lenses have different aperture size (4 mm and 10 mm). The reason why we adopted the LC lens with small aperture size (4 mm) is because projected beam size is around 4 mm depending on size of LCoS panel and the designed optical system. Actually, the concept we proposed in the manuscript could be extended to other AR systems and then the aperture sizes of LC devices should be enlarged. The polarizer-free LC lenses with large aperture size could be achieved by adopting a multilayered approach^26^.

**Figure 5:**
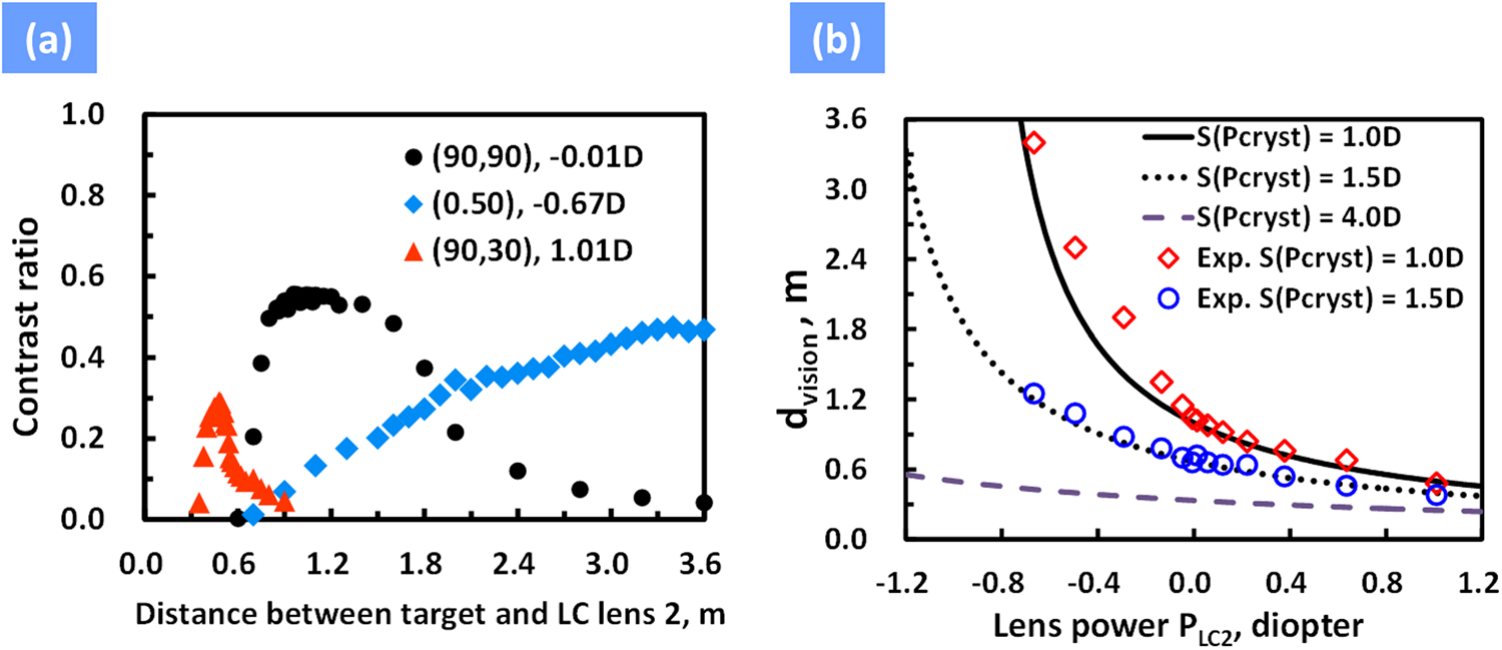
(**a**) Contrast ratio as the function of distance between target and “LC lens 2” at different voltage pair(*V*_1_, *V*_2_) of “LC lens 2”. (**b**) *d*_*vision*_ as the function of lens power of “LC lens 2” at different *S(P*_*cryst*_).

To demonstrate the concept of the proposed AR system in Fig. 1, we construct the system by putting all components together. The focal length of a concave mirror was 30.94 mm. A camera (EOS, 500D Canon) was used to simulate human eyes. Here we turned off “LC lens 1” and LCoS panel. In experiments, we set the range of effective lens power of human eye (i.e. camera here) *S*(*P*_*cryst*_) is in a range of 1D ~ 1.5D (i.e. accommodation of 0.5D), which means the corresponding range of clear vision is 100 cm to 66.7 cm. To test the vision correction function by using “LC lens 2”, the camera was placed right behind the “LC lens 2” and we adjusted camera in order to see the object located 100 cm away from “LC lens 2” which simulates the condition of *S*(*P*_*cryst*_) = 1D. Next we put the target at different distance (z) away from “LC lens 2” and the target was a displayed panel (Surface Pro 4, Microsoft) with a resolution chart. The taken images of the resolution chart were further calculated as contrast ratio (CR) defining as (*I*_max_ − *I*_min_)/(*I*_max_ + *I*_min_), where *I*_max_ and *I*_min_ are the maximum and minimum of the intensity. Contrast ratio as a function of distance z at different applied voltage pairs of “LC lens 2” (*V*_1_, *V*_2_) is shown in Fig. 5(a). In Fig. 5(a), the maximum contrast ratio at a fixed lens power of “LC lens 2” represents the image in focus and the peak distance denotes *d*_*vision*_. In Fig. 5(a), *d*_*vision*_ changes from 3.4 m to 0.48 m with the lens power of “LC lens 2” from −0.67D to 1.01D. At zero lens power of “LC lens 2” (black dots in Fig. 5(a)), *d*_*vision*_ is 1 m. Under the help of the lens power of “LC lens 2”, *d*_*vision*_ could be extended to 3.4 m and to 0.48 m. “LC lens 2” indeed can correct vision by the electrically tunable lens power. According to Fig. 5(a), we then plotted d_vision_ as a function of lens power of “LC Lens 2”(*P*_*LC2*_) at different *S*(*P*_*cryst*_), as shown in Fig. 5(b). According to Eq. (1) and the lens power of “LC lens 2” in Fig. 4, *d*_*vision*_ should be 50 cm~303 cm (i.e. 1/(1 + 1.01) m~1/(1 − 0.67) m), which is similar to experimental results. The theoretical calculations and experimental results are also plotted in Fig. 5(b). In addition, the range of *d*_*vision*_ could be further extended by adding ability of accommodation of eyes. For example, *d*_*vision*_ is 0.48 m to 3.4 m at *S*(*P*_*cryst*_) of 1D in Fig. 5(b); however, the *d*_*vision*_ could be extended to 0.25 m when the eye has ability of accommodation of 3D (i.e. *S*(*P*_*cryst*_) = 4D).

**Figure 6:**
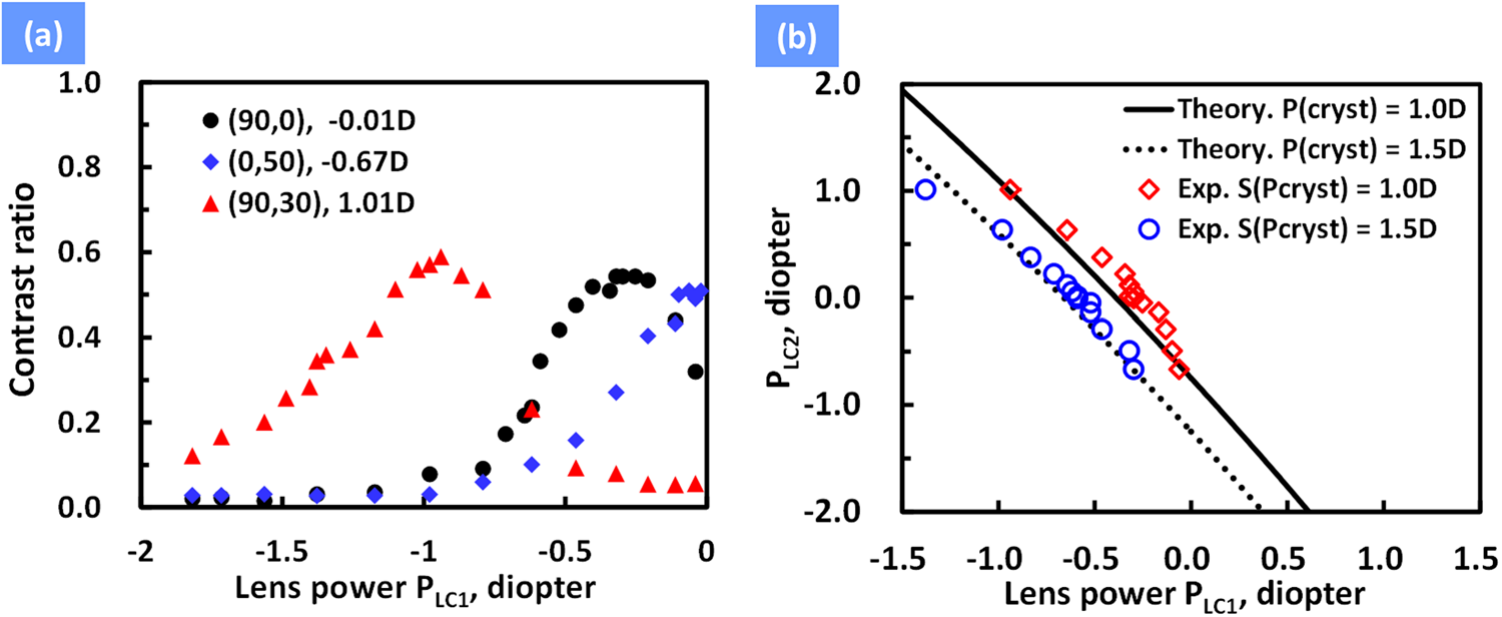
(**a**) The contrast ratio as a function of applied voltage *V*_1_ of “LC lens 1” at *V*_2_ = 75 V_rms_ (2 kHz) at different lens power of “LC lens 2”. (**b**) Lens power of “LC lens 2”(*P*_*LC*2_) as a function of lens power of “LC lens 1”(*P*_*LC*1_) for *S*(*P*_*cryst*_) = 1D and 1.5D. Circles and diamonds stand for experimental results. Black solid line and dotted line stand for theoretical calculation.

Since the vision correction of “LC lens 2” has been demonstrated, we next turn on “LC lens 1” and LCoS panel to further demonstrate both of the functions of vision correction and registration. The resolution chart with spatial line pairs input to the LCoS panel were projected as a virtual image to the camera with same setting as previous one (i.e. *S*(*P*_*cryst*_) = 1D~1.5D). According to results in Fig. 5(b), a given *d*_*vision*_ with a corresponding *P*_*LC*2_ is known. We chose a fixed *d*_*vision*_ (i.e. the object distance in focus) by proper lens power of “LC lens 2”. Thereafter, we projected the virtual image and the location of virtual image changes at different lens power of “LC lens 1”. To measure the location of virtual images (i.e. *d*_*image*_ in Eq. (3)), we took photos at different lens powers of “LC lens 1” by adjusting voltages. Do the same analysis of contrast ratio of photos. The contrast ratio of the virtual image as a function of lens power *P*_*LC*1_ at a given *P*_*LC*2_ is shown in Fig. 6(a). The peak contrast ratio means the situation that *d*_*vision*_ = *d*_*image*_. In Fig. 6(a), the *d*_*image*_ theoretically changes from 51 cm to 282 cm as the voltage pair (*V*_1_, *V*_2_) of “LC lens 1” changes from (26 V_rms_, 75 V_rms_) to (56 V_rms_, 75 V_rms_). This indicates the location of virtual images is electrically adjustable by using “LC lens 1” which solves the registration problem. Moreover, both of the vision correction and registration are achieved in the proposed AR system. Furthermore, we investigate the relation between lens powers of two LC lenses (i.e. *P*_*LC*1_ and *P*_*LC*2_). We replotted the results in Fig. 6(a) as *P*_*LC*2_ as a function of *P*_*LC*1_ in Fig. 6(b). We also plotted the calculation results based on Eq. (4) with measured parameters of *d*_1_ = 3.75 cm, *d*_2_ = 6 cm, and *d*_*system*_ = 3.08 cm. The experimental results are closed to calculations. This indicates that *P*_*LC*1_ and *P*_*LC*2_ are correlated to each other when both registration and vision correction are achieved simultaneously. Typically, the lens powers of LC lenses with large aperture ranges from −1.5 D to +2.0 D^26^. The viewing distance is assumed from 32 cm to infinity. This means the range of operating lens powers for “LC lens 2” and *S(P*_*crystal*_), where the augmented reality can perform, is from −3.84D to 3.16D according to Eqs (2) to (6). To enlarge the range of the viewing distance, lens powers of LC lenses should be enlarged and d_system_ should be reduced.

**Figure 7:**
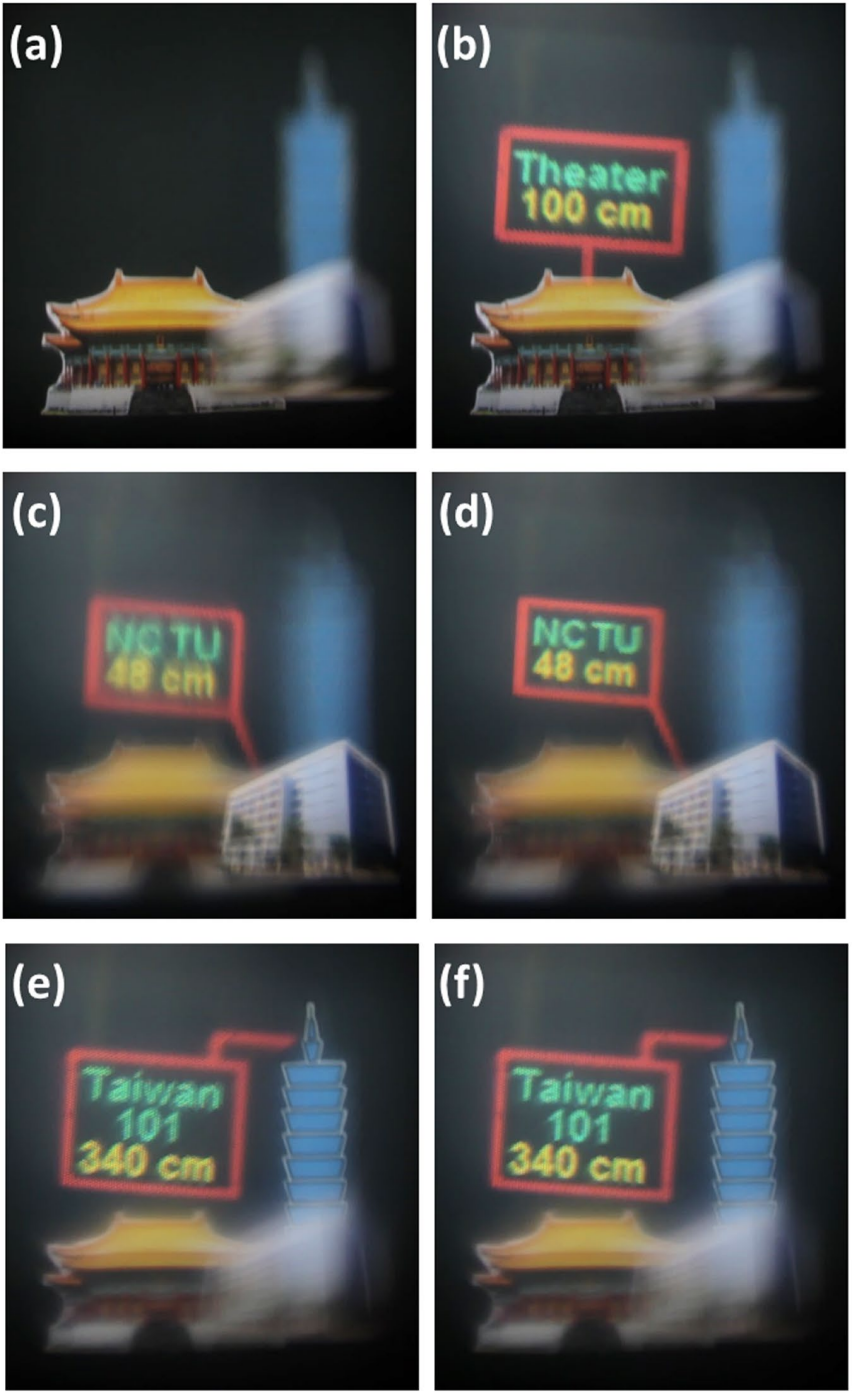
Images of proposed AR system with vision correction and registration function. Three printed objects: “Taiwan 101 building”, a “theater”, “NCTU building” were placed at the distance of 340 cm, 100 cm, 48 cm in front of “LC lens 2”. (**a**)The camera was set to see the object at 100 cm in focus when two LC lenses and LCoS panel were off. (**b**) The virtual image was aligned with theater when we turned on LCoS panel with input of a text and added lens power of −0.25 D of “LC lens 1”. (**c**) from (**b**), to correct presbyopia, lens power of 1.01 D of “LC lens 2” was added. But the virtual image at 100 cm was blurred. (**d**) From(**c**), change the lens power of “LC lens 1” to −0.94 D. Both of NCTU building and the virtual image were clear.(**e**) from (**b**), to correct Myopia, lens power of −0.67 D of “LC lens 2” was added. But the virtual image at 100 cm was still blurred. (**f**) from (**e**), change the lens power of “LC lens 1” to −0.10 D. Both of Taiwan 101 building and the virtual image were clear. The movie file is in ref. 25. The “ NCTU building” is imaged by Pi Cheng Wu and the copyright is released to the authors and Nature Publishing Group. The “Theater” is photographed by Fred Hsu and distributed under license of CC BY-SA 3.0 (https://creativecommons.org/licenses/by-sa/3.0/) on https://en.wikipedia.org/wiki/National_Theater_and_Concert_Hall,_Taipei#/media/File:Taiwan_2009_Taipei_National_Theater_at_Chian_Kai_Shek_Cultural_Center_FRD_7363_Pano_Extracted.jpg.

Here, we demonstrate the images taken from the proposed AR system. The camera was set to see the object when the object distance is 100 cm away from the “LC lens 2”, which are aimed to simulate human eyes with myopia and presbyopia. Three printed objects: “Taiwan 101 Building”, a “theater”, “NCTU building” were placed at the distance of 340 cm, 100 cm, 48 cm in front of “LC lens 2” as the real objects. When the LCoS panel and two LC lenses were turned off, the camera saw the theater clearly, but blurring images of other objects, as shown in Fig. 7(a). When the LCoS panel was turned on, the projected virtual image of “Theater 100 cm” was aligned with the real object of the theater at zero lens power of “LC lens 2”(i.e. *P*_*LC*2_ = 0) and lens power of “LC lens 1” is −0.25 D by applying (*V*_1_, *V*_2_) = (48 V_rms_, 75 V_rms_) at *f* = 2 kHz, as shown in Fig. 7(b). To correct presbyopia, the lens power of +1.01D of “LC lens 2” was added by applying (*V*_1_, *V*_2_) = (90 V_rms_, 30 V_rms_) at *f* = 3.75 kHz. As a result, “NCTU building” located at 48 cm was in focus, as shown in Fig. 7(c). However, the virtual image of “NCTU 48 cm” was blurred because it was projected at 100 cm. To align the virtual images with the theater, we added lens power of −0.94 D of the “LC lens 1” by applying (*V*_1_, *V*_2_) = (26 V_rms_, 75 V_rms_) at *f* = 2 kHz. The virtual image was then aligned well with NCTU building, as shown in Fig. 7(d). Similarly, to correct myopia, we added lens power of −0.67 D of the “LC lens 2” by applying (*V*_1_, *V*_2_) = (0, 50 V_rms_) at *f* = 0.4 kHz. The 101 building was then in focus, but the virtual image at 100 cm was blur, as shown in Fig. 7(e). Both of Taiwan 101 building and the virtual image could be clear once we added lens power of −0.10 D of “LC lens 1” by applying (*V*_1_, *V*_2_) = (56 V_rms_, 75 V_rms_) at *f* = 2 kHz, as shown in Fig. 7(f). In Fig. 7(c) and (e), the lens powers of “LC lens 2” are different and both of virtual images were at 100 cm. However, it seems virtual image in Fig. 7(c) is obscurer than that in Fig. 7(e). This is because the depth-of-field of “LC lens 1” increases with image distance, as well as the depth-of-focus also increases with object distance^29^. Moreover, one could watch the recorded movie files in the link of refs 30 and 31. To sum up, the proposed AR system could solve the challenges of vision correction and registration by using two LC lenses. From Fig. 7, we estimated the tunable range of the lens power of “LC lens 1” is approximately 0.84D for the virtual image from 48 cm to 340 cm. Enlarging the tunable lens power of “LC lens 1” could further enlarge the tunable registration range of the virtual image, for example, from 25 cm to 500 cm. As for “LC lens 2”, the tunable lens power should be at least 4D (i.e. 25 cm to infinite). To achieve this, adopting higher birefringence of LC and adding more LC layers are the ways to go. To have a delicate virtual image, the higher resolution LCoS panel can be adopted.

**Figure 8:**
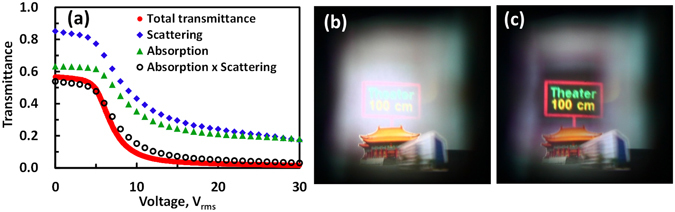
The electro-optics property of the attenuator and images taken with from an environment with strong ambient light. (**a**) The transmittance as a function of applied voltage at *D* = 15 cm at *λ* = 532 nm. Red line stands for the total transmittance. The blue diamonds represent the experiment results of transmittance due to light scattering. The green triangles represent the experiment results of transmittance due to light absorption. The hollow circles represent the calculated total transmittance from light absorption and light scattering. Images was recorded under a strong ambient light when (**b**) the LC attenuator was off and (**c**) the LC attenuator was on (30 V_rms_; *f* = 1 kHz). An unpolarized light was used. The “ NCTU building” is imaged by Pi Cheng Wu and the copyright is released to the authors and Nature Publishing Group. The “Theater” is photographed by Fred Hsu and distributed under license of CC BY-SA 3.0 (https://creativecommons.org/licenses/by-sa/3.0/) on https://en.wikipedia.org/wiki/National_Theater_and_Concert_Hall,_Taipei#/media/File:Taiwan_2009_Taipei_National_Theater_at_Chian_Kai_Shek_Cultural_Center_FRD_7363_Pano_Extracted.jpg.

Final challenge is readability of virtual images under strong ambient light, and the proposed AR system could overcome it by adopting a polarizer-free and electrically tunable attenuator. The total transmittance measured directly as the detector was located at *D* = 15 cm is shown in the red line of Fig. 8(a). When the applied voltage exceeds a threshold voltage (~4.5 V_rms_), the transmittance decreases with applied voltage is because of an increase of absorption and scattering^27^. The highest transmittance is approximately 57% at *V* = 0. The ratio of highest transmittance at 0 V_rms_ to lowest transmittance at 30 V_rms_ is around 40:1. Two factors affect the transmittance of the LC attenuator: one is scattering from polymer networks and the other is light absorption from dye molecules. To quantitatively estimate the absorption and scattering, we assume the light intensity (*T*) after light passes through the LC attenuator is $T={T}_{0}\times {e}^{-\alpha (V)\cdot x}\times {e}^{-\beta (V)\cdot D}$ according to Beer’s law, where *T*_0_ is the initial light intensity impinging to the LC attenuator, *α*(*V*) is the absorption coefficient, *β*(*V*) is the scattering coefficient, x is the thickness of the LC attenuator, d is the distance between the LC attenuator and the detector. Based on experiments (Supplementary Information S1 and Fig. S1), the estimated results of *e* ^−*β*(*V*)·*D*^ (blue diamonds) multiplied by *e* ^−*α*(*V*)·*x*^ (green triangles) are plotted in hollow circles of Fig. 8(a), which are closed to the measured results (the red line). In Fig. 8(a), the transmittances at *V* = 0 contributed from absorption and scattering are 63 and 85%, respectively. The transmittances at 30 V_rms_ contributed from absorption and scattering are 18 and 17%, respectively. As a result, the absorption from dye molecules is larger than the scattering at *V* = 0. The absorption and scattering contribute similarly at high voltage.

Thereafter, we placed the LC attenuator to the AR system as shown Fig. 1(a). We turned off the LC attenuator and set the condition as described in Fig. 7(b), but enhanced the ambient light. As one can see in Fig. 8(b), the virtual image (3.4 mW/cm^2^) was washout under strong ambient light (195.3 mW/cm^2^). We then applied 30 V_rms_ (*f* = 1 kHz) to the LC attenuator to enlarge the light absorption and scattering. By darken the ambient light (108.9 mW/cm^2^) that was shone on the virtual image, the contrast ratio of the virtual image is better, as shown in Fig. 8(c). Scattering could affect the clarity of the vision when people see the environment; however, scattering could help the virtual image projected to eyes. Therefore, the LC attenuator in AR system is capable of solving image washout under strong illumination.

In Fig. 7(b) to (f), the image performance of AR system is good. However, the dispersion of LC materials could lead to chromatic aberration. We also measured wavefronts of “LC lens 2”and then converted wavefronts into lens powers at different wavelengths of 445.6 nm, 543.5 nm, and 633 nm as an example. The “LC lens 2” was applied (*V*_1_, *V*_2_) of (90 V_rms_, 30 V_rms_) at which reached maximum positive lens power in Fig. 4. The measured lens powers of “LC lens 2” are + 1.29D in 446.5 nm, +1.03D in 543.5 nm, and +0.90D in 633 nm (Supplementary Information S2 and Fig. S2). The lens power changes ~0.4D in visible region. To reduce the chromatic aberration, LC materials with low dispersion should be developed. In addition, polarization independency could affect the image quality^26^. The asymmetry of electric fields across to two LC layers could affect polarization independency. Instead of the bottom planar electrode and the hole-patterned electrode, the LC lenses adopted two hole-patterned electrodes could help to improve electric fields more symmetrically across to two LC layers^28^. The central polymer layer is optically isotropic where the molecules of polymer layer were perpendicular to glass substrate because of high applied voltage (300 V_rms_) during fabrication. As the result, the polymer layer does not reshape the electric field; therefore, electric field pattern does not affect by polymer layer.

## Conclusion

An augmented reality (AR) system is demonstrated for the first time to solve three challenges: vision correction, registration, and readability under strong ambient light. The aim is to develop AR device for bionic people. Our system could be a prototype for AR system, where the structure can be redesign with other alternative elements, such as the beam splitters and LC lens be replaced by grating and liquid lens respectively^32, 33^. The polarizer-free LC attenuator can be replaced by the light shutter, such as optical elements made in photo-chromatic materials or thermochromic materials^2, 34^. In AR system, special ray tracing technique, light field technique, and 3D integral imaging technique could be included to even achieve immersive image performances for versatile applications^35–40^. Actually, the LC lenses suffer from limitation of materials, aperture size, polarization dependency, response time, and image quality^41^. Recently, many research groups demonstrated LC lenses to solve challenges of polarization dependency, limitation of aperture size, response time, and even to achieve performance of diffraction-limit^18, 23, 26, 42–44^. LC lenses for ophthalmic applications and hard contact lenses are also developing^18, 26, 45–49^. LC lenses are blooming in applications of visual optics and still waiting for more tests in human subject.

## Methods

### Optical Set-up of Augmented Reality System

The details of the system in Fig. 1(a) are as follows. The system consists of a light source (i.e. LED; TouchBright X3 TB-X3-RCP, LCI), a reflective display (i.e. liquid-crystal-on-silicon panel or so-called LCoS panel, Himax Display Inc. HX7033, resolution of 320 × 240 pixels), a polarizer, a polarizing beam splitter (PBS), a beam splitter (BS), two LC lenses (“LC lens 1” and “LC lens 2”), a concave mirror, and a LC attenuator.

### Fabrication of LC lenses

The structures of two LC lenses are shown in Fig. 2(a) and (b). The structures of two lenses were similar which mainly consist of 3 layers of glasses, two layers of ITO sheet electrode, a layer of 10 mm hole-patterned ITO electrode, an insulting layer (NOA81), one high resistive layers with resistance of 10^6^ ohm/sq (a mixture of PVA and conductive polymer), a layer of PI, two alignment layers (PVA and PI), a polymeric layer with identical alignment ability in both sides, and two LC layers (LN3; Provided by Prof. Liang Xiao, Tsinghua University, *Δn* = 0.369 for *λ* = 589.3 nm at 20 °C). The two LC layers in “LC lens 1” were identical, but the orientations of LC molecules of two LC layers in “LC lens 2” were orthogonal, as illustrated in Fig. 2(a) and (b). The orientations of two LC layers were set by alignment layers and polymeric layers. The fabrication process of a polymeric layer was as follows. The materials of polymeric layers were a mixture of nematic LC (Merck, MLC 2144, *Δn* = 0.2493 for *λ* = 589.3 nm at 20 °C), reactive mesogen (Merck, RM-257), and a photoinitiator (Merck, IRG-184) with a 20:79:1 wt% ratio. The mixture was filled into an empty cell consisting of two ITO glass substrates coated with alignment layers which were mechanically buffered either anti-parallel (“LC lens 1”) or orthogonal directions (“LC lens 2”). The thickness of the empty cell was 35 μm. Thereafter, the cell was applied an AC voltage of 300 V_rms_ with a frequency of 1 kHz and then exposed to UV light with intensity of 3 mW/cm^2^ for an hour for polymerization. After polymerization, a glass substrate was peeled off and then the polymeric layer with thickness of 35 μm was obtained.

### Fabrication of LC Attenuator

The structure of the LC attenuator is shown in Fig. 3(a), where the cell gap between two glass substrates with coated ITO electrode was 5 μm. The polyimide (PI) layer *without* rubbing treatment was coated above ITO electrode to provide vertical alignment for the LC directors. The middle LC layer with polymer network was fabricated with injection of a mixture which was composed of nematic LC ZLI-4788 (Merck, ne = 1.6567, *Δn* = 0.1647 at *λ* = 589 nm; *Δε* = −5.7 at *f* = 1 kHz), a diacrylate monomer (bisphenol-A-dimethacrylate), and a dichroic dye S428 (Mitsui, Japan) at 90:5:5 wt% ratios. Then, followed by shining the UV light (*λ*~365 nm, *I*~3 mW/cm^2^), the cell was cured at a fixed temperature of 19.5 degree Celsius for 1.5 hr. After photo-polymerization, the polymer networks were grown along the z direction since the LC directors were aligned perpendicular to the glass substrates during the UV curing process.

### Measurement of lens power of LC lenses

The lens powers of two lenses were measured with a Shack-Hartmann wavefront sensor (Thorlabs, WFS150-7AR). The experimental setup consisted of light source (laser diode, *λ* = 543 nm), a single mode fiber, a solid lens, a pair of solid lenses to construct a 4-f system, and a wavefront sensor. Light source was coupled into the fiber, and the light came out from another end of the fiber can be regarded as a point light source. Then the point source coupled to a lens generated a collimated light. The collimated light was impinged on sample (i.e. LC lens), 4-f system, and wavefront sensor, accordingly. The sample was placed in front of the 4-f system at the distance of the focal length of the first lens of 4-f system; the wavefront sensor is placed behind the 4-f system at the distance of the focal length of the second lens of 4-f system. The wavefront generated by sample (LC lens) was relayed to the wavefront sensor and then is recorded. For polarization-dependent sample (i.e. “LC lens 1”), we added an extra polarizer to the sample. The wavefront (*W*) or spatial optical path differences (OPDs) recorded by wavefront sensor were expended by Zernike polynomials^50^: $W=\sum _{i=0}{c}_{i}{Z}_{i}$, where *c*_*i*_ and *Z*_*i*_ represent the Zernike coefficients and Zernike polynomials, respectively. The lens power of measurement wavefront (*P*) was defined as the sphere power (*S*) plus half of the cylinder power (*C*), and it related to the defocus coefficient c_4_ as.

10 $$P=S+0.5C=-4\sqrt{3}\times {c}_{4}/{R}^{2},$$

where *R* was the radius of the aperture we measured (*R* = 4 mm). In the measurement, we attached a pinhole of 4 mm in diameter on “LC lens 1” because of the effective aperture of “LC lens 1” in the AR system is 4 mm.

### Measurement of contrast ratio of real image (“LC lens2”)

The measurement of *d*_*vision*_ was based on the proposed AR system in Fig. 1. To test the vision correction function by using “LC lens 2”, the camera (EOS, 500D Canon) was placed right behind the “LC lens 2” and we adjusted camera in order to see the object located 100 cm away from “LC lens 2” which simulates the condition of *S*(*P*_*cryst*_) = 1D. Next, we turned off the LCoS panel and put the target at different distance (z) away from “LC lens 2”; the target was a displayed panel (Surface Pro 4, Microsoft) with a resolution chart. By using software Matlab, we analyzed the recorded intensity of the resolution chart. The contrast ratio (CR) of a spatial line pair in the resolution chart is defined as (*I*_max_ −* I*_min_)/(*I*_max_ +* I*_min_), where *I*_max_and *I*_min_ are the maximum and minimum of the intensity, respectively. Contrast ratio as a function of distance z at different applied voltage pairs of “LC lens 2” (*V*_1_, *V*_2_) is shown in Fig. 5(a). In Fig. 5(a), the maximum contrast ratio at a fixed lens power of “LC lens 2” represents the image in focus and the peak distance denotes d_vision_.

### Measurement of contrast ratio of virtual image (“LC lens1”)

The measurement of *d*_*image*_ was based on the proposed AR system in Fig. 1. The resolution chart with spatial line pairs input to the LCoS panel were projected as a virtual image to the camera with same setting as previous one (i.e. *S*(*P*_*cryst*_) = 1D). First, the camera (EOS, 500D Canon) was placed right behind the “LC lens 2” and we chose a fixed *d*_*vision*_ (i.e. the object distance in focus) by a proper lens power of “LC lens 2”. Thereafter, we projected the virtual image and the location of virtual image changed at different lens power of “LC lens 1” and then took photos of those projected virtual image. By using software Matlab, we also analyzed the recorded intensity of the resolution chart to calculate contrast ratio. The contrast ratio of the virtual image as a function of lens power *P*_*LC*1_ at a given *P*_*LC*2_ is shown in Fig. 6(a). The peak contrast ratio means the situation that *d*_*vision*_ = *d*_*image*_.

### Measurement of electro-optical properties of the LC attenuator

The measured voltage dependent transmittance is plotted in Fig. 8(a) using an unpolarized light (He-Ne Laser; Melles Griot, 05-LGR-173, 5 mW) at *λ* = 543.5 nm.

## Electronic supplementary material

Supplemenary information
