# Supplementary material for: Augmented reality with image registration, vision correction and sunlight readability via liquid crystal devices
Source: Sci Rep. 2017 Mar 27;7:433. doi: 10.1038/s41598-017-00492-2 (PMC5428510; doi:10.1038/s41598-017-00492-2)
Supplement: Supplementary file 1 — Supplemenary information [file 41598_2017_492_MOESM1_ESM.pdf]

Supplementary Information for

Augmented reality with image registration, vision correction and sunlight readability via liquid crystal devices

Yu-Jen Wang[1,3], Po-Ju Chen[1,3], Xiao Liang[2] and Yi-Hsin Lin[1,*]

[1] Department of Photonics, National Chiao Tung University, Hsinchu, Taiwan R.O.C

[2] Department of Chemistry, Tsinghua University, Beijing, China

[3]These authors contributed equally to this work

*Corresponding author: [*] yilin@mail.nctu.edu.tw

http://web.it.nctu.edu.tw/~yilin/

This PDF file includes:

S1: Optical properties of optical attenuator: scattering and absorption

S2: Chromatic aberration of a LC lens: wavefronts at different wavelengths

Supplementary Information S1. Optical properties of optical attenuator: scattering and absorption.

After light passes through the LC attenuator, we assume the light intensity (T) is $T = T_0 \times e^{-\alpha(V)\cdot x} \times e^{-\beta(V)\cdot D}$ according to Beer's law, where T_0 is the initial light intensity impinging to the LC attenuator, $\alpha(V)$ is the absorption coefficient, $\beta(V)$ is the scattering coefficient, x is the thickness of the LC attenuator, *D* is the distance between the LC attenuator and the detector. In the experiment, a collimated laser light with the wavelength of 543.5nm (Melles Griot, Model 05-LGR-173) impinged to the LC attenuator and a detector (New Focus, Model 2031) was used to collect the light intensity after the LC attenuator. To measure the scattering effect, the detector was placed at different distance (d) away from the LC attenuator. The transmittance as a function of the applied voltage (V) at different *D* is plotted in Fig. S1(a). The transmittance decreases with voltage when applied voltage exceeds a threshold voltage (~4.5 V_{rms}). In addition, the transmittance decreases as the distance of the detector increases at a fixed voltage. This is because of light absorption originating from dichroic dye molecules and the light scattering resulting from polymer networks. The light absorption does not depend on distance of d; however, light scattering depends on distance of *D*. To further analysis the scattering effect, we plotted transmittance as a function of distance of *D* in Fig. S1(b) based on the results in Fig, S1(a). Thereafter, we fitted the results of Fig. S1(b) based on an exponential fitting in order to obtain the $\beta(V)$. Since the light absorption is independent of d, we plotted transmittance of $e^{-\beta(V)\cdot D}$ contributed from light scattering at *D*=15 cm in Fig. S1(c) (blue diamonds). To measure the transmittance from light absorption, we measured the voltage-dependent transmittance of $e^{-\alpha(V)\cdot x}$ at *D*=0 and the transmittance is mainly contributed from absorption from dye molecules, as shown in Fig. S1(c)(green triangles). From Fig. S1(c), we also calculated $\alpha(V)$: $\alpha(0V_{rms}) = 0.091\mu m^{-1}$ and $\alpha(30V_{rms}) = 0.342\mu m^{-1}$. The total transmittance measured directly as the detector was located at *D*=15 cm is shown in the red line of Fig. S1(c). The calculated results of $e^{-\beta(V)\cdot d}$ multiplied by $e^{-\alpha(V)\cdot x}$ is plotted in hollow circles of Fig. S1(c) which is closed to the measured results (the red line). As a result, we conclude that two factors affect the transmittance of the LC attenuator: one is scattering from polymer networks and the other is light absorption from dye molecules. At low voltage, absorption contributes more. At high voltage, the scattering and absorption contribute near equally. Scattering could affect the clarity of the vision when people see the environment; however, scattering could help the virtual image projected to eyes.

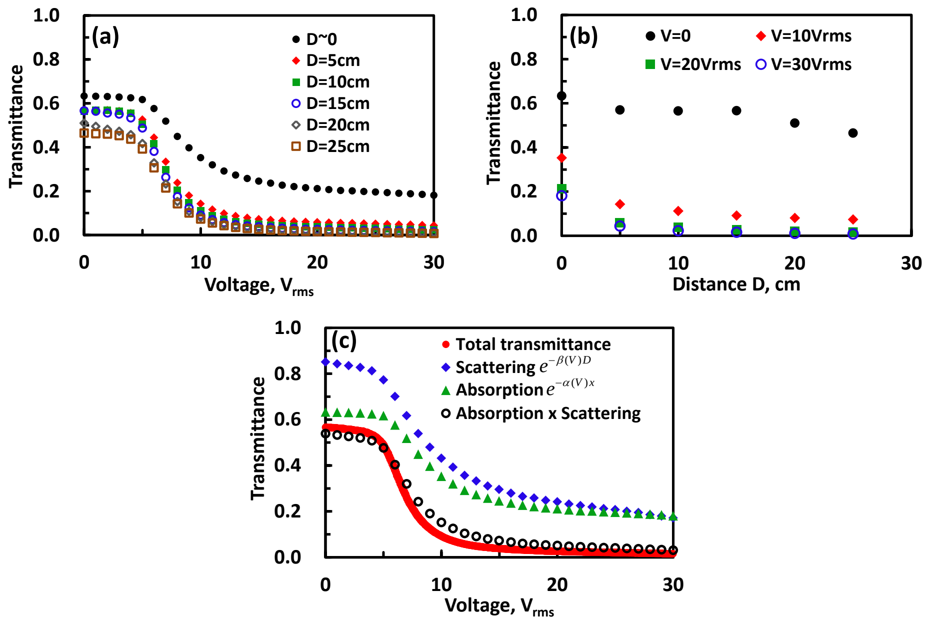

Fig. S1. Transmittance of the LC attenuator. (a) The transmittance as a function of applied voltage at different distance *D*. (b) is replotted from (a) for transmittance v.s. distance *D* at different applied voltages. (c) The transmittance as a function of applied voltage at *D*=15cm. Red line stands for the total transmittance. The blue diamonds represent the experiment results of $e^{-\beta(V)\cdot D}$ from light scattering. The green triangles represent the experiment results of $e^{-\alpha(V)\cdot x}$ from light absorption. The hollow circles represent the calculated total of $e^{-\beta(V)\cdot D}$ multiplied by $e^{-\alpha(V)\cdot x}$.

Supplementary Information S2. Chromatic aberration of LC lens: wavefronts at different wavelengths

The wavefronts after a plane wave propagated in "LC lens 2" were measured by a Shack-Hartmann wavefront sensor at wavelengths of 446.5nm, 543.5nm, and 633nm, respectively. The "LC lens 2" applied (V_1, V_2) of ($90V_{rms}$, $30V_{rms}$) at 3.75 kHz reached maximum positive lens power in Fig. 4. The measured results of wavefront are shown in Figs. S2(a) to (c). The different wavefronts at differences wavelength is because the dispersive properties of LC materials resulting in the discrepancy in optical phase at different wavelengths. From Figs. S2(a), (b), and (c), we replotted optical path difference along y pupil coordinate of 0 v.s. x-pupil coordinate at different wavelengths in Fig. S2(d). The curves in optical path difference changes with the wavelengths. By converting the wavefront into lens power, the lens powers are +1.29D in 446.5nm, +1.03D in 543.5nm, and +0.90D in 633nm. To improve the chromatic aberration, we can adopt LC materials with low dispersion.

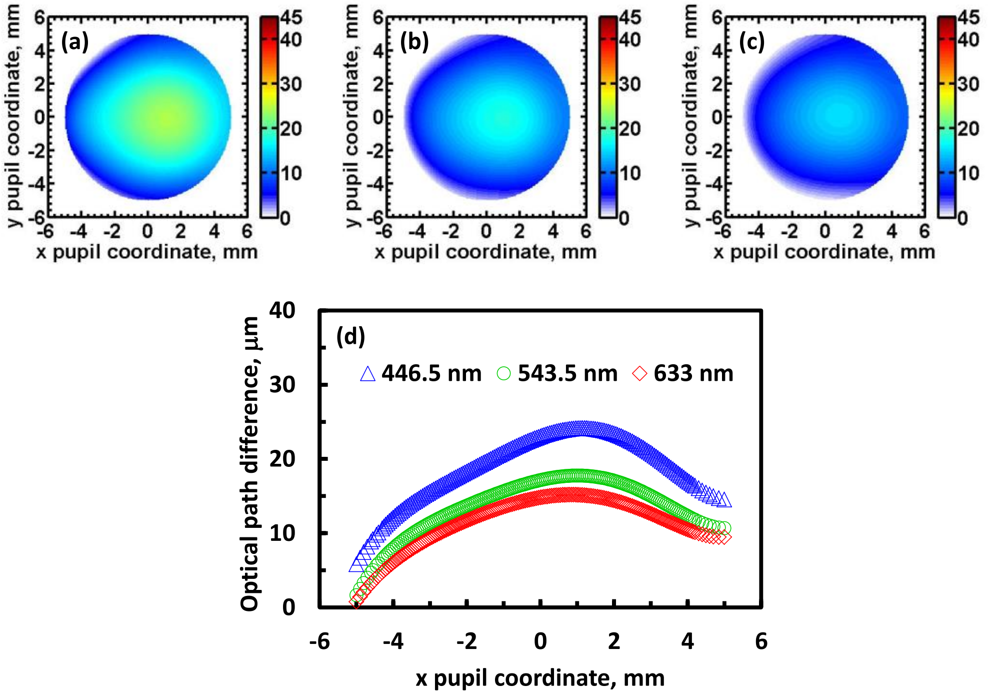

Fig. S2. The wavefronts of "LC lens 2" under voltage pairs (V_1,V_2) of ($90V_{rms}$, $30V_{rms}$) at wavelengths of (a) 446.5 nm, (b) 543.5 nm, and (c) 633 nm. Color bar indicates optical path difference in a unit of micron. (d) From (a), (b), and (c), we replotted optical path difference v.s. x-pupil coordinate at different wavelengths.
